# Identification of a strawberry flavor gene candidate using an integrated genetic-genomic-analytical chemistry approach

**DOI:** 10.1186/1471-2164-15-217

**Published:** 2014-04-17

**Authors:** Alan H Chambers, Jeremy Pillet, Anne Plotto, Jinhe Bai, Vance M Whitaker, Kevin M Folta

**Affiliations:** 1 Horticultural Sciences Department, University of Florida, Gainesville, FL, USA; 2 Horticultural Research Laboratory, Agriculture Research Service, USDA, Ft. Pierce, FL, USA; 3 Gulf Coast Research and Education Center, University of Florida, Gainesville, FL, USA; 4 Plant Molecular and Cellular Biology Program, University of Florida, Gainesville, FL, USA

**Keywords:** RNA-seq, γ-decalactone, *Fragaria*, Flavor, Molecular marker, Strawberry

## Abstract

**Background:**

There is interest in improving the flavor of commercial strawberry (*Fragaria* × *ananassa*) varieties. Fruit flavor is shaped by combinations of sugars, acids and volatile compounds. Many efforts seek to use genomics-based strategies to identify genes controlling flavor, and then designing durable molecular markers to follow these genes in breeding populations. In this report, fruit from two cultivars, varying for presence-absence of volatile compounds, along with segregating progeny, were analyzed using GC/MS and RNAseq. Expression data were bulked *in silico* according to presence/absence of a given volatile compound, in this case γ-decalactone, a compound conferring a peach flavor note to fruits.

**Results:**

Computationally sorting reads in segregating progeny based on γ-decalactone presence eliminated transcripts not directly relevant to the volatile, revealing transcripts possibly imparting quantitative contributions. One candidate encodes an omega-6 fatty acid desaturase, an enzyme known to participate in lactone production in fungi, noted here as *FaFAD1*. This candidate was induced by ripening, was detected in certain harvests, and correlated with γ-decalactone presence. The *FaFAD1* gene is present in every genotype where γ-decalactone has been detected, and it was invariably missing in non-producers. A functional, PCR-based molecular marker was developed that cosegregates with the phenotype in F_1_ and BC_1_ populations, as well as in many other cultivars and wild *Fragaria* accessions.

**Conclusions:**

Genetic, genomic and analytical chemistry techniques were combined to identify *FaFAD1*, a gene likely controlling a key flavor volatile in strawberry. The same data may now be re-sorted based on presence/absence of any other volatile to identify other flavor-affecting candidates, leading to rapid generation of gene-specific markers.

## Background

The commercial strawberry (*Fragaria* x *ananassa*) (2*n =* 8*x =* 56) is a popular fresh and processed fruit with substantial value worldwide. It is recognized for its sweet flavors and appealing aromas. The volatile profiles of strawberry are relatively complicated among berries, with over 360 volatile compounds reported [1]. A reduced set of approximately 20 volatiles are commonly reported to be important components of strawberry flavor [2-4]. The principle flavor compounds include esters [5], ketones, terpenes [6], furanones [7], aldehydes [8], alcohols, and sulfur-containing compounds. The concentrations of individual volatiles are highly dependent on species [9], environment and harvest date [2], [10-12], cultivar, postharvest treatment and fruit developmental stage [13].

One important volatile compound is γ-decalactone (γ-D; CAS 706-14-9). This volatile is described as “fruity”, “sweet”, or “peachy” [9] and contributes to fruit aroma [14,15]. The volatile tends to be undetectable in some genotypes [2], while in others its accumulation varies greatly within and between harvest seasons [12]. This pattern suggests that a critical biosynthetic step or substrate may be missing or limited, and under strong environmental influence. The high variability may be due to differences in expression of genes encoding enzymes linked to the process. The observation that some genotypes never produce the compound when others do presents an excellent basis to use global transcriptome profiling to identify candidate genes associated with is production or stability. Because the commercial strawberry is octoploid, F_1_ progeny from a volatile producer and a non-producer have led to predictions about inheritance of a given volatile [2,16].

A number of researchers have used genomics approaches to identify marker-trait associations in polyploids using SNPs. Advances in marker discovery have been made in allohexaploid wheat (*Triticum* spp.) cultivars using the Illumina GoldenGate Assay [17], allohexaploid oat (*Avena sativa* L.) using Roche 454 sequencing [18], and allotetraploid oilseed rape (*Brassica napus*) using Illumina Solexa sequencing [19]. Other approaches have focused on developmental changes in the transcriptome associated with ripening to identify gene candidates. This strategy was effective for grape (*Vitis* spp.) using Illumina sequencing [20], and recently in peach (*Prunus persica* L.) using microarrays [21].

The goal of this work is to use a transcriptome-based approach to identify the genes required for γ-D production. The approach leverages the presence/absence nature of γ-D from specific genotypes, its predictable inheritance, environmental lability, and variation during the growing season. The analysis identified one transcript from a narrow set of gene candidates that is functionally related to genes implicated in biosynthesis of this compound in certain fungi [22-24] and the related compound γ-dodecalactone [25]. A PCR-based amplicon corresponding to the candidate sequence co-segregates with the volatile in a breeding population, corresponding backcrosses, and in select cultivars and wild accessions. We demonstrate that computational bulking of RNAseq data based on the presence or absence of a volatile can identify transcripts likely playing a direct role in volatile production.

## Results

The gene segregating with the presence of the γ-D volatile has been shown to segregate as a single dominant locus, making it a prime candidate for the approach outlined in Additional file 1: Figure S1. Briefly, a cross was constructed between Elyana, a γ-D producing cultivar, and Mara des Bois, a cultivar where γ-D has not been detected. Progeny were grown, and fruits from each individual plant were analyzed for volatiles and coincident gene expression. The fruits from each plant were analyzed and sequenced separately so that transcriptomes from producers and non-producers could be bulked computationally, with the hypothesis that candidate genes would be common to producers, while being expressed low levels or go undetected in non-producers. Results could be experimentally validated in the parental lines and in segregating progeny using gene expression analysis.

### γ-D quantity is genetically and environmentally influenced

**Figure 1:**
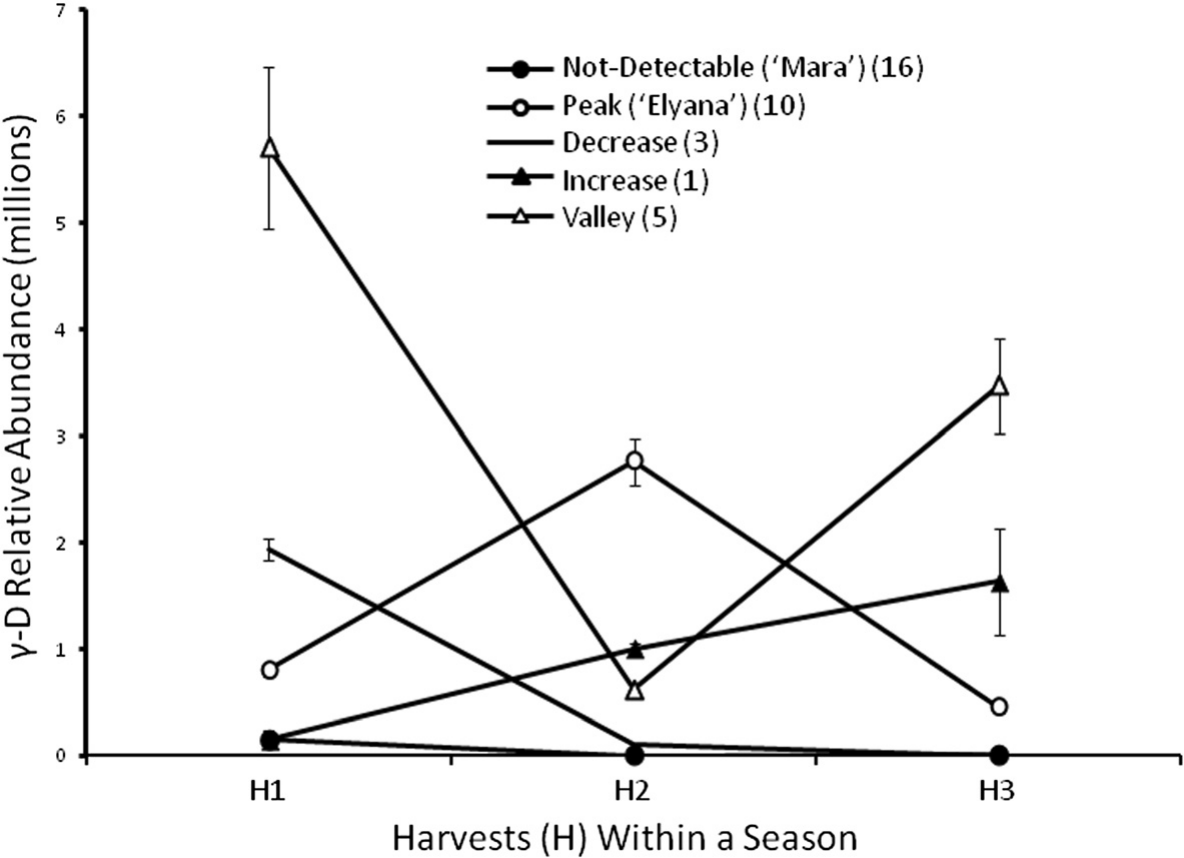
**Representative genotypes showing γ-decalactone stability over a single growing season.** 35 out of 130 ‘Elyana’ x ‘Mara des Bois’ progeny produced fruit suitable for volatile analysis over three consecutive harvests. All progeny could be clustered into five classes based on γ-decalactone production as shown by five uniquely shaped graphs below. ‘Mara des Bois’ represents the class of non-producers, with ‘Elyana’ representing those lines that peaked mid-season. Other patters included the inverse of ‘Elyana’ with a “valley” pattern, a strong “decrease” after the earliest harvest, and one progeny that showed an increase as the season progressed. Counts in each category are shown in the attached legend. Data are from two technical replicates from one example genotype per class. Error bars represent standard deviations.

The first tests examined γ-D accumulation in the ‘Elyana’ and ‘Mara des Bois’ parental lines and representative progeny over a growing season, using detection by GCMS. Genotypes were assayed for γ-D production on three harvest dates. The data are presented in Figure 1, showing data for a single genotype representing each of five general trends. Approximately 50% of the progeny produced no γ-D, similar to ‘Mara des Bois’. The largest portion of the γ-D producers followed a similar trend to ‘Elyana’, with higher amounts in the second harvest compared to the first and third harvests. The reciprocal trend was observed in five genotypes that showed less γ-D during the second harvest compared to the other two harvests. Three genotypes examined produced the highest amount in the first harvest, yet levels remained low the second and third harvests. A single genotype exhibited higher levels as the season progressed. These same volatile patterns were also observed in backcross progeny during the 2012/13 season (data not shown). While there is an approximately three-fold difference in accumulation in the ‘Elyana’ background over the season, no γ-D was ever detected in ‘Mara des Bois’ above background noise.

### γ-Decalactone estimation

**Figure 2:**
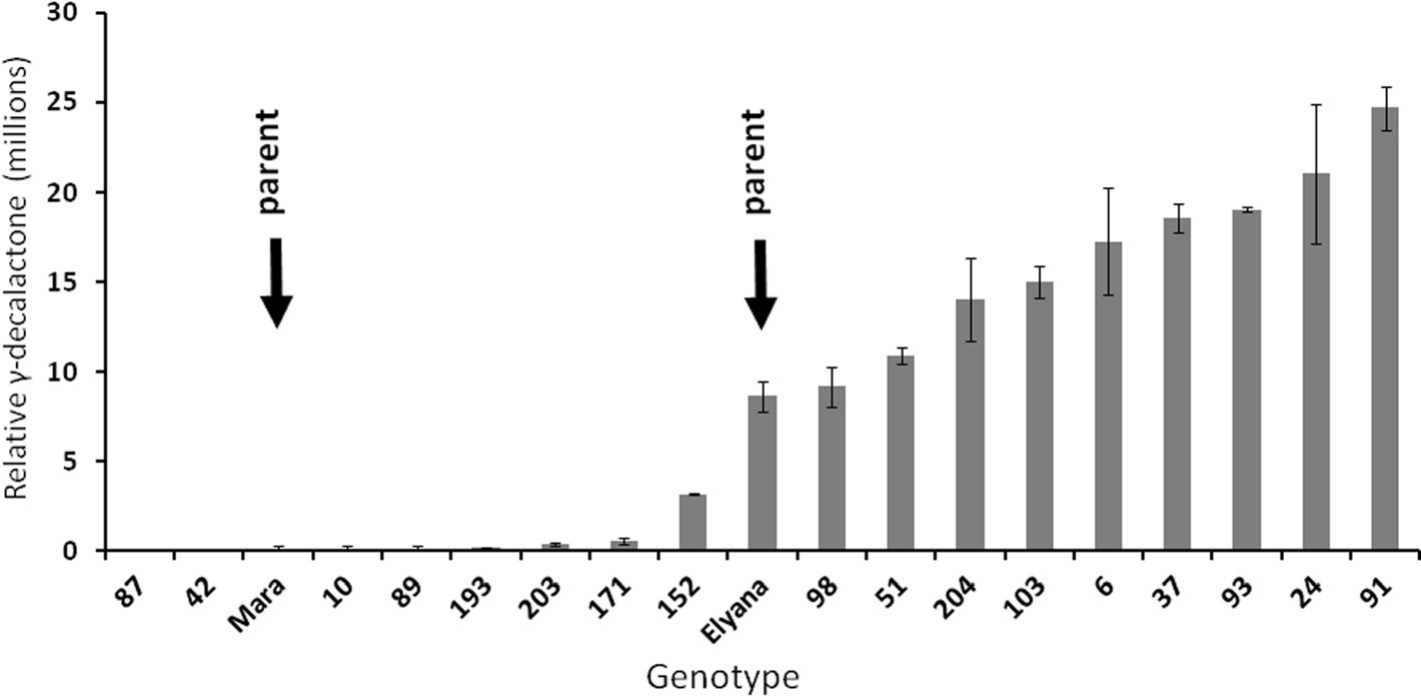
**γ-Decalactone production in a selection of progeny from the ‘Elyana’ x ‘Mara des Bois’ cross.** Total volatiles were analyzed by GC/MS. A number of progeny produced more γ-decalactone than ‘Elyana’ during the harvest shown. A subset of the progeny, along with the ‘Mara des Bois’ parent, never produced γ-decalactone above background levels. Ripe fruit samples from some of these genotypes were split between volatile analysis and RNA-seq transcriptome analysis. Data are from two technical replicates. Error bars represent standard deviations.

The levels of γ-D were estimated by comparing amounts detected in berries from the population using GCMS, with standards derived by adding the pure volatile to half-ripe strawberry fruit. Figure 2 shows the γ-D volatile phenotype for a subset of the ‘Elyana’ x ‘Mara des Bois’ progeny. The top producing 30 genotypes from the 2012/13 season ranged from 0.018 to 0.035 mM γ-D (data not shown).

### Transcriptome profiling

Fourteen progeny and both parents were individually analyzed by RNA-seq. The γ-D non-producers included in this analysis included ‘Mara des Bois’, 42, 89, 193, and 203. The producers were ‘Elyana’, 6, 24, 37, 51, 91, 93, 98, 103, 152, and 204. Many of the producers had higher γ-D levels than the ‘Elyana’ γ-D positive parent (Figure 2). The transcriptomes from individual lines were computationally pooled based on “producer” or “non-producer”. Over 106 million MID-tagged RNAseq reads were generated from each of the parents and progeny. The average number of filtered and mapped reads per genotype was 5.5 million per genotype and ranged from 3 million to 8.5 million. Both parents had more than 7 million filtered, mapped reads.

Approximately 17,000 out of ~31,000 annotated genes in the strawberry draft genome [26] were represented in the RNA-seq dataset. A cursory SNP search identified over 1.7 million SNPs total when compared to the *F. vesca* genome (SNP criteria: 95% minimum *P* not ref, 10 or greater read depth, and present in at least two of the sixteen genotypes’ datasets).

### Gene expression trends in parental lines

**Figure 3:**
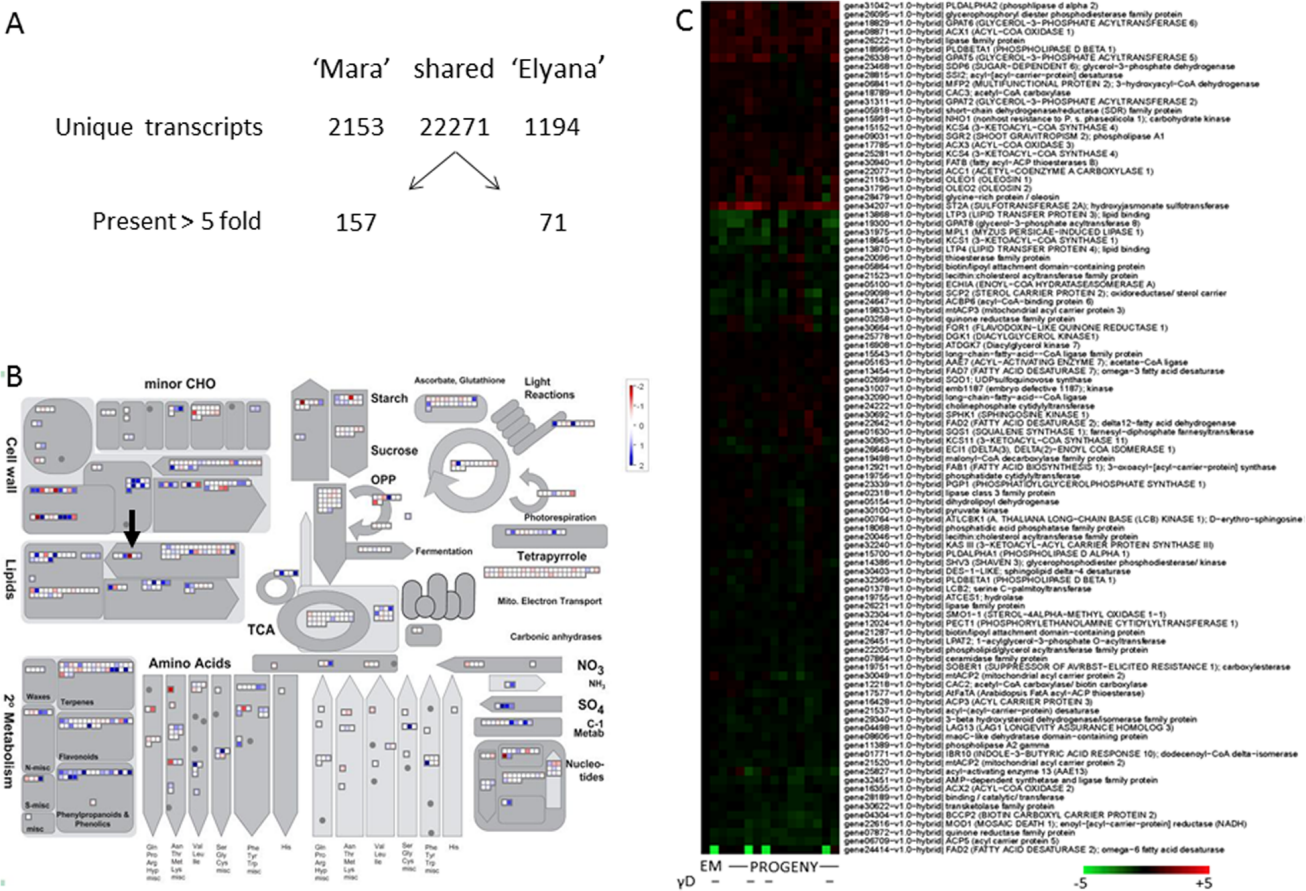
**Differential transcript accumulation in parental genotypes. A**. Unique transcripts detected in each parent as well as those shared between lines. The number of transcripts detected >5 fold is shown for each parent. **B**. MapMan distribution of differentially expressed transcripts separated by GO terms. **C**. Transcript accumulation from genes associated with “lipid” annotation in ‘Elyana’ (E), ‘Mara des Bois’ (M) and segregating progeny. The minus sign (-) indicates the inability to detect γ-decalactone in those lines.

Alignment of all reads against the diploid *F. vesca* genome produced transcript assemblies that provided cursory detail of gene expression difference between the two parental genotypes. Comparisons of differentially-expressed transcripts, including transcripts with low RPKM representation, between the ‘Mara des Bois’ and ‘Elyana’ parents showed that among the 23,718 transcripts predicted from assembly of all reads, 2,153 were unique to the ‘Mara des Bois’ parent and 1,194 were only observed in ‘Elyana’ (Figure 3, Panel A). When transcripts composed of higher RPKM values were compared there were 157 that had a 5-fold or greater abundance in ‘Mara des Bois’ and 71 that had a >5-fold abundance in ‘Elyana’. When grouped by GO function the differentially expressed genes show no clear pattern (Figure 3B) favoring any one category.

Table 1 shows twelve transcripts that were the most abundant in the γ-D producing parent. The highest expressed was an omega-6-fatty acid desaturase transcript (gene24414), followed by a transcript annotated as osmotin stress/defense (gene32423). The set also includes two serine-threonine protein kinases (gene09445 and gene00774), citrate synthase (gene26778), an F-box protein (gene12328), a proline transport protein (gene21705), and several uncharacterized, hypothetical proteins.

**Table 1:** Transcripts abundant in gamma-decalactone-producing genotypes

**Featured ID**	**Seq. Description**	**GO biological process**	**GO cellular component**	**GO molecular function**
gene24414	Omega-6 fatty acid endoplasmic reticulum isozyme 2-like	Unsaturated fatty acid biosynthesis process; oxidation-reduction process;	Endoplasmic reticulum membrane; integral to membrane;	Delta12-fatty acid dehydrogenase activity; oxidoreductase activity, acting on paired donors, with oxidation of a pair of donors resulting in the reduction of molecular oxygen to two molecules of water; omega-6 fatty acid desaturase activity;
gene32423	Osmotin-like protein osm34	Defense response to bacterium, incompatible interaction; response to salt stress; defense response to fungus, incompatible interaction;	#N/A	#N/A
gene26993	NA	#N/A	#N/A	#N/A
gene09445	Serine threonine-protein kinase rio1-like	Phosphorylation;	#N/A	ATP binding; protein serine/threonine kinase activity;
gene26778	Citrate synthase	Tricarboxylic acid cycle; cellular carbohydrate metabolic process; response to cadmium ion;	Cell wall; mitochondrial matrix; chloroplast;	Zinc ion binding; citrate (Si)-synthase activity; ATP binding;
gene14440	NA	#N/A	#N/A	#N/A
gene13132	PREDICTED: uncharacterized protein in LOC101300103	#N/A	#N/A	#N/A
gene05924	atp-dependent-nad h-hydrate dehydrate-like	#N/A	#N/A	#N/A
gene12328	f-box protein at5g07610-like	#N/A	#N/A	#N/A
gene21705	Diphthamide biosynthesis protein 2-like	Proline transport;	#N/A	#N/A
gene00774	Serine threonine protein phosphatase	#N/A	#N/A	Hydrolase activity;
gene23527	Uncharacterized loc101207862	Generation of precursor metabolites and energy;	Chloroplast; membrane; mitochondrion;	#N/A

Transcripts here represent those that are correlate best with accumulation of gamma-decalactone (Pearson’s p < 0.001).

### Computational bulking to limit candidate set

The large number of differentially-expressed transcripts could be further narrowed by analyzing transcript patterns for these genes in progeny segregating for γ-D. Pairwise comparisons were made between genotypes with high (genotypes ‘Elyana’, 91, 24, 37, 103, and 006) and non-detectable (genotypes ‘Mara des Bois’, 203, 89, and 42) γ-D levels. Gene candidates were filtered to have a modest >4-fold increase in transcript support of producers over non-producers. Using this approach, a single gene candidate was identified, gene24414 on linkage group 3 (LG3:31112418..31114643, scf0513029:129621..131846), the same abundant transcript shown in Table 1 as variable between the two parents. To illustrate how the integration of segregating progeny can separate out transcripts not common to γ-D volatile producers, all transcripts from the “lipids” category are shown in Figure 3C from the parental genotypes and several of their progeny. In general, lipid related transcripts show limited differential accumulation between any genotypes. The clear exception is the omega-6-fatty-acid desaturase (bottom row) which is not detected in ‘Mara des Bois’ (M) and in three of the progeny (green squares). The γ-D volatile was not detected in these same genotypes. Of all candidates from Table 1, the omega-6-fatty-acid desaturase was the only transcript that correlated 100% with the ability to produce γ-D. The gene was given the designation *FaFAD1*. A 1,128 bp open reading frame was cloned from ‘Elyana’ cDNA (Additional file 2).

### Validation of key candidates

**Figure 4:**
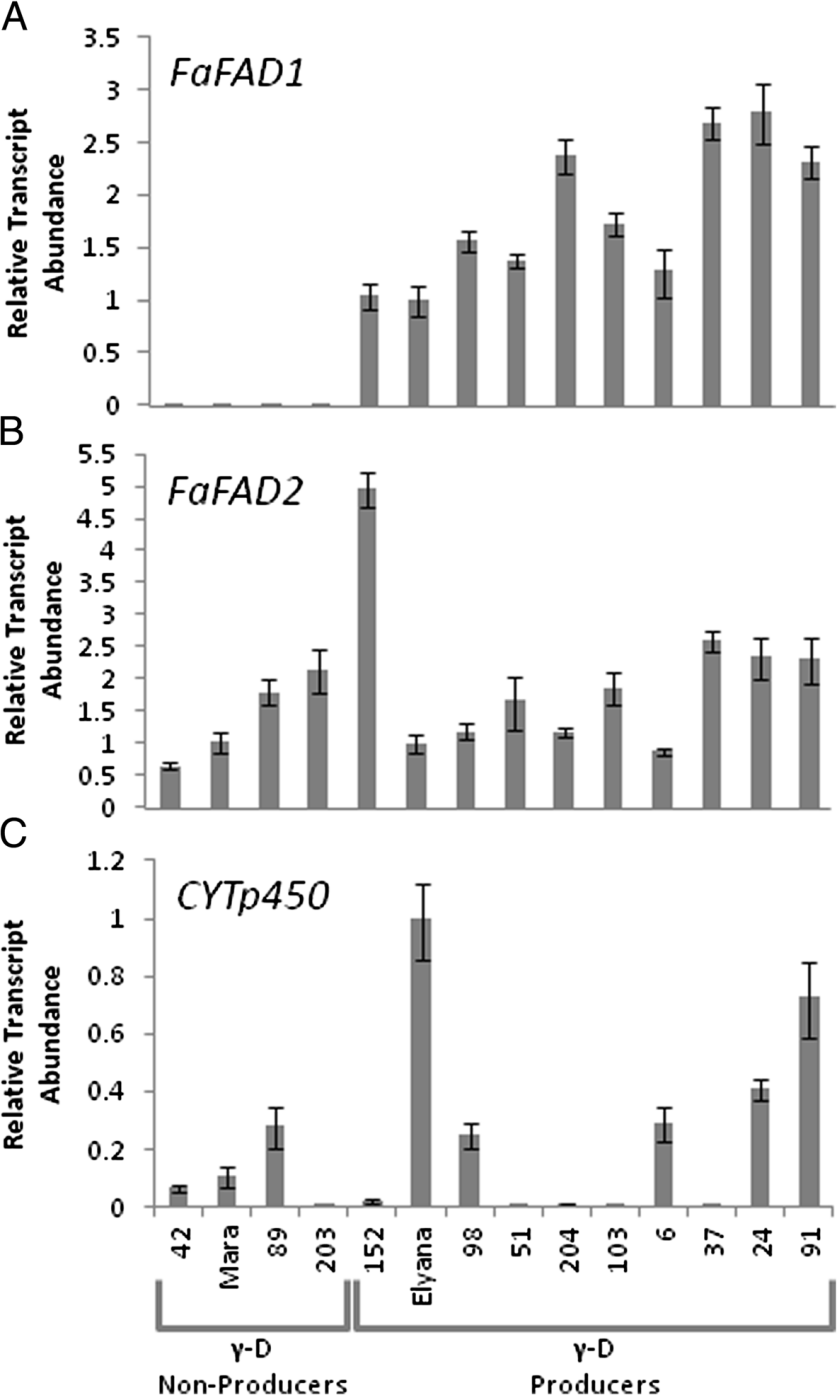
**qRT-PCR results from three gene candidates correlated with the γ-decalactone phenotype.** A single gene, *FaFAD1* (gene24414), **(A)** encoding a putative ω-6-fatty acid desaturase was identified as differentially expressed between high and low γ–decalactone genotypes. Another putative fatty acid desaturase, *FaFad2* (gene22642), was found in the *F. vesca* genome and is shown in **(B)**. Reducing the stringency by reducing the number of progeny in each phenotypic pool resulted in another candidate **(C)***CYTp450* (gene29958), a putative cytochrome p450 monoxgenase, located in proximity to *FaFAD1*. qRT-PCR results are shown for each of these genes using ‘Elyana’ as the comparator against a subset of progeny. Data are from three technical replicates with error bars representing standard deviations.

The steady-state transcript accumulation of FaFAD1 (Figure 4A), gene22642 (FaFAD2; Figure 4B), and gene29958 (a cytochrome p450 oxidase termed FaCYTp450; Figure 4C) was tested. The results for *FaFAD2* and *FaCYTp450* are not consistent with the ability to produce γ-D, and were therefore de-prioritized as candidates. The qRT-PCR results for *FaFAD1* visually correlated more closely with the volatile phenotype than the RNA-seq RPKM values. Both methods were similar in failure to detect transcript support for *FaFAD1* in γ-D non-producers.

### Candidate genes in fruit developmental series

**Figure 5:**
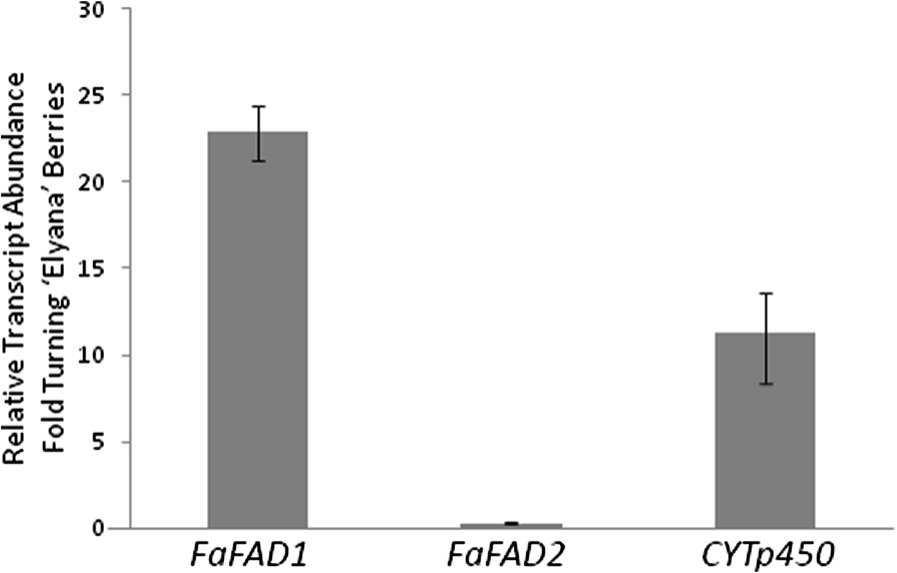
**qRT results for γ-decalactone gene candidates tested against an ‘Elyana’ developmental fruit series.** Three stages of fruit were tested (green expanding, blushing, and full red, ripe). The γ-decalactone phenotype is only detectable in fully ripe fruit. Only comparisons between blushing and ripe developmental stages are shown. The *FaFAD1* and *CYTp450* genes show ~21-fold and ~11-fold (respectively) ripening induction in ‘Elyana’. Data are from three technical replicates with error bars representing standard deviations.

An ‘Elyana’ fruit series was tested for ripening induction of *FaFAD1*, *FaFAD2*, and *FaCYTp450* as shown in Figure 5. The fold-change in transcript abundance for each gene is shown for ripe fruit compared to blushing fruit. *FaCYTp450* showed a >11-fold increase (+/- 0.30) in transcript abundance between ripe and blushing fruit. *FaFAD1* showed a >21-fold increase (+/- 0.33) in transcript abundance. *FaFAD2* was not ripening induced. These results were consistent over at least three independent biological replicates.

### Candidate genes and two environments

**Figure 6:**
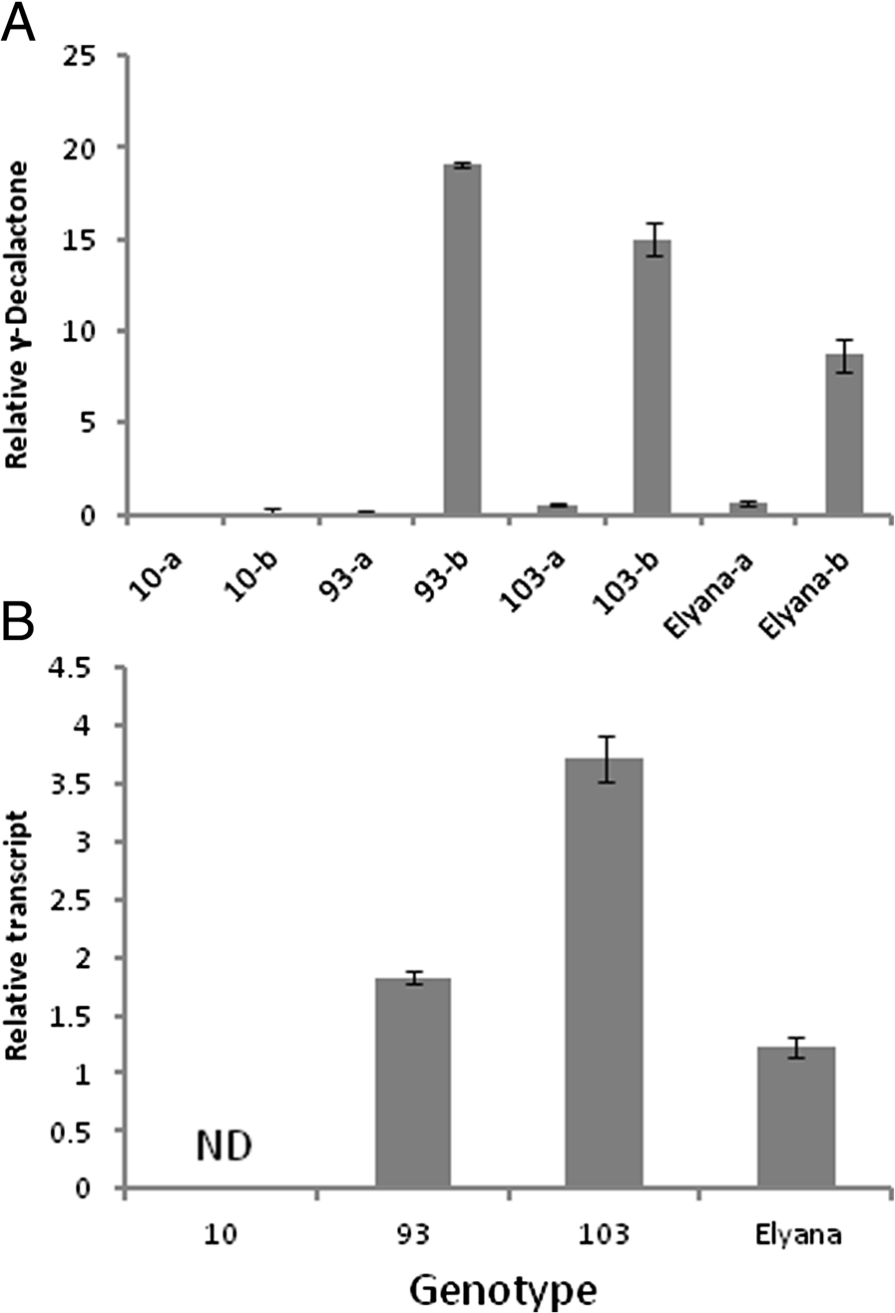
**Presence of γ-decalactone in non-inductive and inductive environments, and their correlation with *FaFAD1 *transcript accumulation.** Comparison of γ-decalcatone detected from environment ‘a’ (non-inductive) compared to environment ‘b’ (inductive) **(A)**. Genotypes where *FAD1* transcript is detected produce the volatile only in inductive environment ‘b’. Others tested (e.g. line 10) did not produce detectable γ-decalactone in either environment. **(B)** The relative *FaFAD1* transcript accumulation for genotypes shown in **(A)**.

Environmental fluctuation of γ-D accumulation is shown in Figure 2. To test if *FaFAD1* matched this pattern, transcript abundance was examined in tissues obtained from two different harvests. The population average for all γ-D producers in environment “a” was approximately eleven-fold less than the population average for γ-D in environment “b”. γ-D non-producers had levels of γ-D only consistent with noise during either harvest, but γ-D producers showed high environmental effects. Figure 6A shows the γ-D accumulation for four genotypes (10, 93, 103, and ‘Elyana’) in these two environments. Figure 6B shows the qRT-PCR results for these genotypes in the two environments when transcript abundance in environment “b” was compared to environment “a”. Only modest increases are shown for genotypes 93, 103, and ‘Elyana’ with genotype 10 (non-producer) showing no evidence for *FaFAD1* transcript accumulation.

### Molecular basis of γ-decalactone loss-of-function

**Figure 7:**
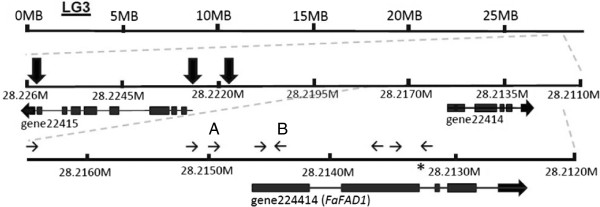
***FaFAD1 *structure.** The *FaFAD1* gene, noted also as gene24414, is located in a region corresponding to linkage group 3 (LG3) in the *F. vesca* genome. Simple sequence repeat regions are noted by the large black arrows, and the small black arrows represent primers used to test for amplification within this region in parental lines and progeny. The letters **A** and **B** denote the primers used for marker amplification. The asterisk denotes a potential allele introducing a stop codon into the *FaFAD1* sequence.

The lack of detectable transcript in the ‘Mara des Bois’ parent and in specific progeny may be due to at least one of two factors. First, that transcription/mRNA accumulation of the candidate *FaFAD1* is blocked. Alternatively the functional gene or allele may be missing altogether. To test these possibilities several primer pairs were designed to amplify the genomic sequence upstream and internal to *FaFAD1*. A map of the genomic region and the corresponding primer pairs is provided in Figure 7. In all cases, none of the primer pairs could amplify products from genotypes unable to produce γ-D, while amplicons across the region were produced from every plant where γ-D was detected.

### γ-decalactone marker in three populations

**Figure 8:**
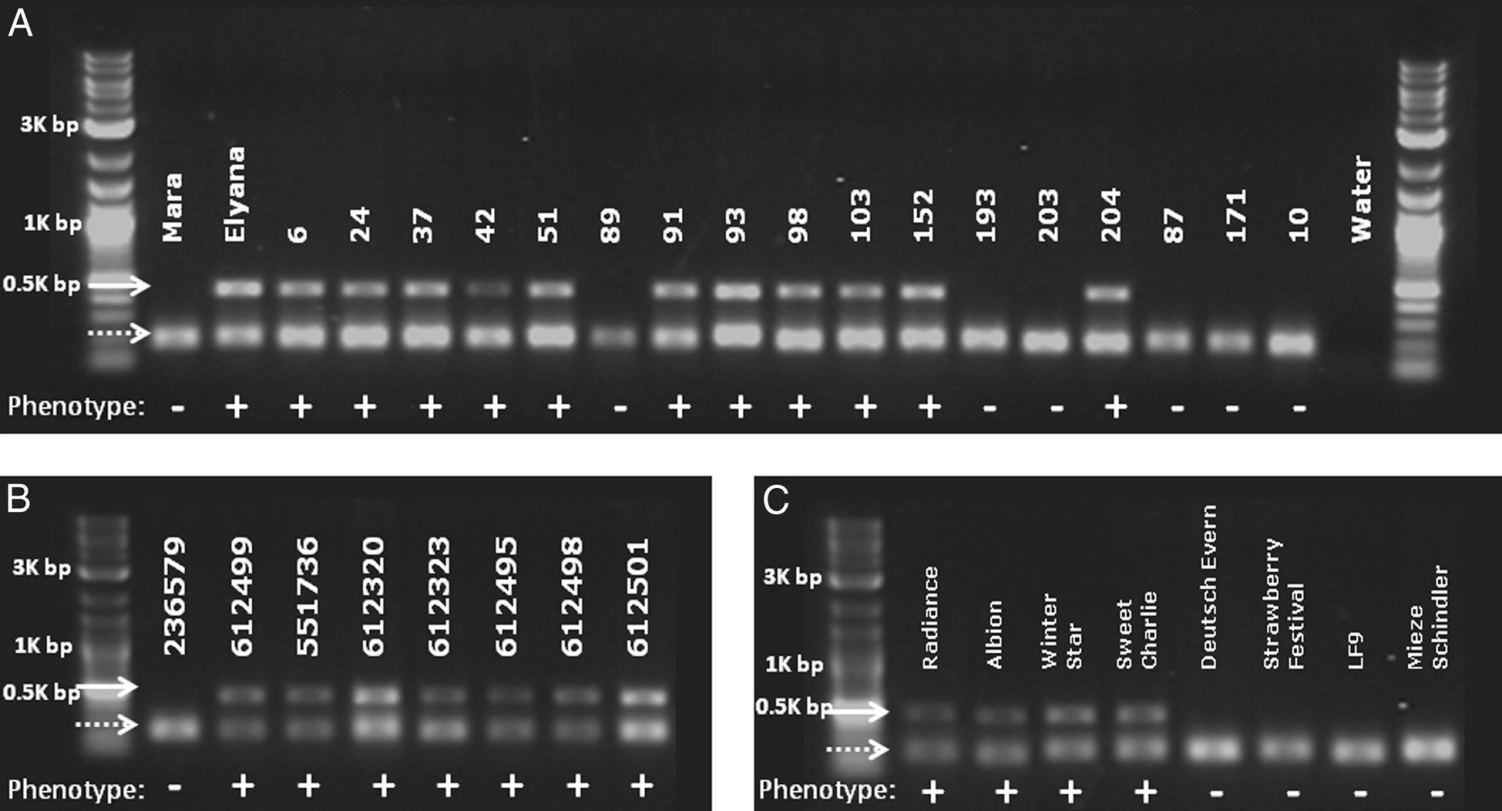
**Co-segregation of the *FaFAD1*, PCR-based marker with the γ–decalactone phenotype.** The *FaFAD1*-based PCR product is denoted by the single arrow and migrates at 500 bp by design. The dashed arrow denotes a positive PCR control (BFACT045) that is located in proximity of *FaFAD1*. Plus and minus signs represent the γ–decalactone phenotype for each genotype as determined by GC/MS. **(A)** The parental lines ‘Elyana’ x ‘Mara des Bois’ are shown with a subset of their progeny. **(B)** A series of wild octoploid materials representing one *F. chiloensis* and seven *F. virginiana* accessions (number = GRIN PI accession). **(C)** The correlation of the PCR product and γ–decalactone phenotype in cultivars unrelated to populations described in this work.

The ability to amplify a product specifically in γ-D producing genotypes provided an opportunity to design a gene-based molecular marker. A γ-D PCR-based assay was designed using *FaFAD1* primers and then tested against three populations. The first was a subset of 19 genotypes from the original ‘Elyana’ x ‘Mara des Bois’ F_1_ population (Figure 8A). The presence of the 500 bp PCR amplicon (solid arrow) was detected exclusively in genotypes shown to produce γ-D. The positive PCR control (BFACT045) is shown by the dashed line arrow. The second and third populations tested were BC_1_ crosses to the ‘Elyana’ and ‘Mara des Bois’ parents with progeny 98 as the male in each case. The ‘Elyana’ backcross contained 26 progeny, each with at least three harvests during the 2012/13 growing season. The marker co-segregated with the phenotype in all cases. Twenty-two progeny in the ‘Mara des Bois’ backcross had at least three volatile harvests during the 2012/13 season. Each cosegregated with the marker and phenotype (data not shown).

The potential molecular marker was also tested against a set of cultivars with demonstrated present or undetectable γ-D. ‘Radiance’, ‘Albion’, ‘Winter Star’, and ‘Sweet Charlie’ were all γ-D positive and positive also for the PCR product (Figure 8C). ‘Deutsch Evern’, ‘Strawberry Festival’, ‘LF9’, and ‘Mieze Schindler’ were all negative for γ-D and also negative also for the marker. ‘Strawberry Festival’ and ‘LF9’ were additionally interesting because ‘LF9’ is a seedling from self-pollination of ‘Strawberry Festival’ [27]. The prediction would therefore be that ‘LF9’ would be negative for the marker and γ-D phenotype. This was confirmed by volatile and marker analysis.

### SSR marker development

**Figure 9:**
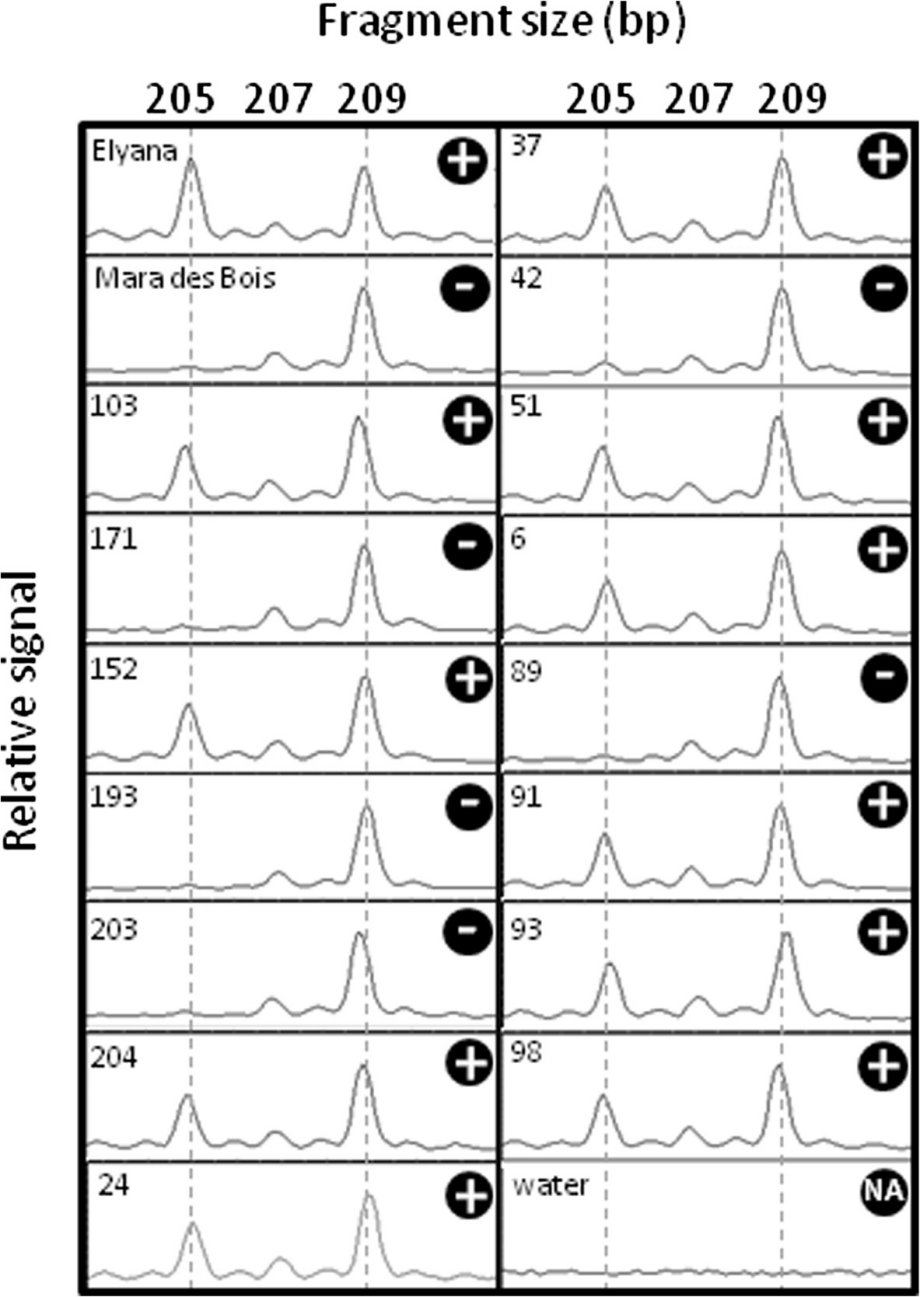
**Cosegregation of the γ–decalactone phenotype and an SSR-based marker 11 kb upstream of *FaFAD1*.** The parents and progeny were tested with primers corresponding to a microsatellite sequence located adjacent to the *FaFAD1* gene. While all genotypes were monomorphic for a PCR product at 209 bp, the 205 bp product was only detected in the ‘Elyana’ parent and any genotype where γ–decalactone was detected, as designated by the “+”.

An SSR marker was developed to investigate cosegregation of alleles more distantly positioned relative to *FaFAD1*. Figure 9 shows the results of the SSR tested in the parents ‘Elyana’ and ‘Mara des Bois’, and 15 progeny selected from both γ-D producers and non-producers. Few progeny were tested because the objective of the SSR marker design was simply to demonstrate the potential for converting a gene candidate into a second type of molecular marker commonly used for *Fragaria* genotyping. ‘Elyana’ exhibited four marker alleles (205, 209, 215, and 219), and ‘Mara des Bois’ only possesses the 209 allele. For clarity, only the 205 and 209 alleles are shown. Allele 209 was monomorphic in all genotypes tested, and alleles 215 and 219 were not associated with the γ-D phenotype. Progeny 103, 152, 204, 24, 37, 51, 6, 91, 93, and 98 were all positive the γ-D phenotype and for allele 205. Progeny 171, 193, 203, 42, and 89 were negative for γ-D and the 205 allele.

## Discussion

Fruit flavor and aroma profiles are shaped by a mixture of sugars, acids and volatile organic compounds. Individual volatile components have been demonstrated to play key roles in consumer liking, as noted in tomato [28], and strawberry [29]. Several reports have detailed the importance of key compounds to aroma production and the genetic loci or genes that control them [6,16]. One component contributing to flavor in strawberry fruits is γ-decalactone (γ-D). This compound represents the recognized aromas in peaches and apricots, and is of interest to industry as a flavoring agent. The synthesis of γ-D is not well-understood in plants. However, several microorganisms can generate γ-D from fatty-acid substrates [30] and are used as bioreactors to produce this flavor compound.

To identify transcripts related to γ-D production, two parents varying for the volatile were crossed. ‘Elyana’ is a large, firm berry, and bred for production in Florida [31]. ‘Mara des Bois’ is smaller and soft, and not used in wide commercial cultivation. Fruits (red, ripe fruits of similar size, age, and environment) were assayed for the volatile and also for transcripts associated with receptacle/achene ripening. The RNAseq data from producers and non-producers were computationally bulked to identify sequences common to each pool (Additional file 1: Figure S1). Strawberry fruit transcriptomes have been examined previously [32-34], and show changes in a substantial number of transcripts throughout the ripening process. The commercial strawberry is octoploid and highly heterozygous [35]. Crossing two plants produces a myriad of progeny phenotypes due to contributions from homoeologous genes. The segregation within subgenomes likely provides additional “noise” that allows for transcripts relevant to the trait to separate clearly from others, focusing the candidate set. Transcriptomes for each plant were considered independently, so the transcriptomes of γ-D producers and non-producers could be compared *in silico*.

The test began with detection of γ-D. The γ-D producers show fluctuations in volatile quantity throughout the growing season (Figure 1). The genotypes with the highest concentrations were estimated to possess between 0.028 to 0.035 mM γ-D (data not shown). Gamma decalactone accumulation was not ever detected in the ‘Mara des Bois’ parent. The range of differences observed in parental fruits and progeny presented an ideal situation to assay global gene expression coinciding with volatile production.

When progeny were sorted by presence/absence of γ-D, a small subset of transcripts was significantly different between phenotypic groups. Pairwise comparisons of RNAseq data around a fruit phenotype resolved to an especially strong single candidate, a transcript encoding an omega-6-fatty acid desaturase (*FaFAD1*). This gene is located near a QTL previously reported to explain about 90% of the γ-D phenotype in strawberry [16]. Another gene recently found in peach (*Pp*FAD*_1B-6*, 71% identity and 82% similar to *Fa*FAD1 at the protein level) was correlated with γ-D in ripening fruit, and demonstrated ω-6 oleate desaturase activity when overexpressed in yeast [21]. Fatty acid desaturases catalyze the formation of double bonds into fatty acyl chains. The report from Sanchez [36], and the biochemistry inferred from protein sequence, each suggest a role for this gene in lactone production, but the precise biochemical steps have not been demonstrated. Future work will examine the role of this transcript in transgenic lines.

*FaFAD1* transcript abundance correlated well with the presence of γ-D. The transcript accumulated with ripening, and only in certain environmental conditions (Figure 5). The ability to produce γ-D segregated as a single dominant locus, consistent with previous reports [16]. A PCR-based survey of materials from the population indicated that the *FaFAD1* genomic sequence could not be amplified from γ-D non-producers, suggesting gene deletion or radical alteration affecting PCR amplification. Eight total combinations using ten distinctly positioned primers were used to amplify regions upstream of and within the *FaFAD1* gene (Shown in Figure 7). In each case, ‘Elyana’ genomic template amplified the expected fragments, but ‘Mara des Bois’ produced no amplicons. This is consistent with the idea that a deletion is responsible for the absence of *FaFAD1*-related sequence in the ‘Mara des Bois’ genome, and this would explain the dominant, single gene effect for the γ-D phenotype. This finding allowed for development of a PCR-based molecular marker to identify genotypes with the potential to produce γ-D. PCR primers corresponding to the 5′ sequence of *FaFAD1* were used to amplify the region shown in Figure 7 (primers A and B). The presence of the PCR product co-segregated precisely with the ability to produce the γ-D, in the parental cross, in progeny, and in backcross populations (Figure 8A).

The nature of the deletion is further exemplified using simple sequence repeat (SSR) markers, tools frequently used to fingerprint strawberry germplasm [37-39]. The availability of the diploid strawberry, *F. vesca*, genome [26] makes it possible to position the microsatellite sequences within the structural context of the strawberry genome. One SSR sequence is located 11 kb adjacent to the *FaFAD1* gene, and polymorphisms would be predicted to segregate with the candidate gene. The results in Figure 8 show that the presence of allele 205 is a reliable predictor of the ability to produce γ-D, at least in the subset of the population evaluated. These data are important because they provide two independent, PCR-based tests can differentiate γ-D producers and non-producers. While not tested outside of the parental genotypes and only in several progeny, this second primer set offers another potential molecular marker that may be used to verify results from the *FaFAD1* sequence. The findings also indicate that the SSR is present in the ‘Elyana’ parent and not the other, suggesting that the missing *FaFAD1* gene may be part of a larger deletion.

The potential utility of this molecular marker was demonstrated when it was applied to a set of unrelated germplasm that was also varying for presence/absence of γ-D. In this case, the PCR product could only be amplified in genotypes demonstrated to produce γ-D. The evaluation was extended to wild germplasm where fruit could be obtained. Even in these distant accessions, the PCR product was only amplified in lines where γ-D was detectable. The putative marker not only works within a population, but likely will work across commercial breeding populations and wild octoploid accessions. Future studies will track the origin of the gene in diploid germplasm in an attempt to reconstruct its origins, as was done with alleles to trace linalool-producing variants of *FaNES1*[6,40].

There are notable limitations to the approach used in this study. The visual correlation between the γ-D phenotype and qRT-PCR results is shown in Figures 4 and 5. This quantitative trend, however, is not consistent in the RNA-seq RPKM data (data not shown). γ-D producers, irrespective of volatile amount produced, could not be distinguished quantitatively, though producers and non-producers were still easily discernible. This suggests that while the RNA-seq approach worked well for the qualitative γ-D phenotype, uncovering quantitative γ-D effects would be very challenging. This further underscores the need to incorporate independent methods (like qRT-PCR) for examining candidate genes in the early stages of discovery.

The development of two independent molecular markers that segregate with the phenotype in a wide range of germplam will permit improved parental selection and rapid screening of progeny possessing the ability to produce γ-D. A simple and robust assay can rapidly eliminate individual plants from breeding populations, saving time, fuel, space and other resources. Most importantly, the marker allows more rapid integration of a fruity volatile into advanced selections.

## Conclusions

The results of this study demonstrate that gene candidates for strawberry fruit traits may be identified by integrating careful phenotyping and transcriptomic analysis with genetics. This approach rapidly reduced the complexity of the octoploid transcriptome down to a single candidate gene. The identified gene, *FaFAD1*, is functionally equivalent to genes involved in γ-D synthesis in fungi and potentially in peach [21]. Gene identification led to the development of a gene-based marker, enabling selection for γ-D producers at the seedling stage. Moving rapidly from candidate to marker has the potential to increase breeding efficiency and reduce the downstream costs associated with maintaining plants lacking a favorable trait. Looking forward, γ-D is only one of many volatiles that can be analyzed with this approach. The same exact dataset may now be re-sorted to identify candidates for other volatiles. The bulk sorting of polyploid transcriptomes is a rapid and cost effective means to identify a testable suite of genes contributing to a given trait.

## Methods

### Plant materials

Parental lines were *Fragaria* x *ananassa* ‘Elyana’ female (γ-D producer) and ‘Mara des Bois’ male (γ-D non-producer). F_1_ progeny from the ‘Elyana’ x ‘Mara des Bois’ cross were clonally multiplied in a Colorado summer nursery in 2010. Two runner tips of each of approximately 130 seedlings were harvested from the nursery and grown at the Gulf Coast Research and Education Center (GCREC) in Wimauma, Florida during the 2010–11 winter season. These plants were evaluated in the field for horticultural qualities such as yield and fruit size and for volatile diversity using GC/MS. A subset of this population was selected based on flavor volatile diversity and superior plant performance. These selections were further clonally multiplied and grown again in the 2011/12 and 2012/13 seasons.

BC_1_ populations were made by crossing one γ-D positive progeny (progeny 98, male) to each parent (female) in 2011. Backcross progeny were increased in the summer nursery as described above. During the 2012/13 season, the progeny were transplanted to the same commercial strawberry growing system in Florida and analyzed for volatiles and other horticultural traits. Other cultivars for marker validation were maintained at GCREC. All other genotypes used in this study were obtained from the Germplasm Resource and Information Network (GRIN) repository in Corvallis, OR, and maintained in the field at GCREC.

### Fruit volatile analysis

All fruiting progeny from the ‘Elyana’ x ‘Mara des Bois’ cross were analyzed for volatiles by GC/MS during the 2010/11 season. Harvest dates were January 20, February 11, February 25, and March 18, 2011. Backcross populations were harvested during the 2012/13 season and were harvested on January 13, January 31, and March 7, 2013. Data from the 2010/11 harvests were used to select genotypes segregating for volatiles of interest. Fruit processing for volatile analysis was conducted as follows. A representative ~25 g sample was collected from five to six fully ripe, clean, and normal-shaped berries from each genotype. The calyx from each berry was removed, and berries were blended with an equal weight of saturated NaCl solution (~35% NaCl in molecular biology grade water). The volatile 3-hexanone was added as an internal standard to a final concentration of 1 ppm prior to blending. Five ml aliquots were dispensed into 20 ml glass vials and sealed with magnetic crimp caps (Gerstel, Baltimore, MD, USA). Two technical replicates were processed for each genotype at each harvest. Samples were frozen at -20°C until analysis by Gas Chromatography/Mass Spectroscopy.

### Gas chromatography/mass spectroscopy (GC/MS)

A 2 cm tri-phase SPME fiber (50/30 μm DVB/Carboxen/PDMS, Supelco, Bellefonte, PA, USA) was used to collect and concentrate volatiles prior to running on an Agilent 6890 GC coupled with a 5973 N MS detector (Agilent Technologies, Palo Alto, CA, USA). Before analysis, samples were held at 4°C in a Peltier cooling tray attached to a MPS2 autosampler (Gerstel). All other volatile sampling and analysis methods were as previously described [15]. The volatile 3-hexanone was used as an internal control. An authentic γ-D standard (Sigma Aldrich, St. Louis, MO, USA) was run under the same chromatographic conditions as berry samples for verification of volatile identify. The area of each γ-D peak was normalized to the peak area of the internal standard, and normalized peak areas were compared between samples.

### Estimation of γ-decalactone in strawberry fruit

A standard curve was made in half ripe fruit of *F.* x *ananassa* ‘Winterstar’ fruit puree to estimate the amount of γ-D. This approach mimics volatile detection in ripe fruit. Half red fruit were processed with saturated NaCl and 3-hexanone as described in Fruit Volatile Analysis above. Pure γ-D (Sigma; St. Louis, MO) was added to puree aliquots from 0.005 to 0.3 mM concentrations, vortexed thoroughly, and then analyzed by GCMS as with all other samples. Baseline γ-D was identified in puree sampled without pure volatile added.

### Combined volatile and RNA-seq tissue

Fruit for volatile and RNA-seq analyses were harvested on December 15, 2011. Fourteen progeny and both parents (‘Elyana’ and ‘Mara des Bois’) were selected to maximize representation of segregating volatiles. Eight to ten fully-ripe fruit were collected from each genotype, cleaned, the calyx was removed, and then split longitudinally. Half of each progeny’s sample was processed for volatile analysis as described above, and the other half was flash frozen in liquid nitrogen and stored at -80°C until RNA extraction. A blank was processed in between each GC/MS sample to minimize cross-sample contamination.

### RNA extraction

Frozen berries were crushed and then ground to a fine powder in a liquid nitrogen-cooled coffee grinder (KitchenAid Blade Coffee Grinder, St. Joseph, MI, USA). RNA was extracted using a modified method [41]. Two grams of fruit powder was used per 5 ml extraction buffer.

RNA was treated with DNase I, RNase-free (Fermentas, Waltham, MA, USA) according to the manufacturer instructions and then cleaned using the Qiagen RNeasy Mini Kit (Qiagen, Valencia, CA, USA). RNA quality was checked on a Bioanalyzer prior to RNA-seq library construction. Each sample was individually barcoded during library construction. Two lanes with eight libraries each were run on an Illumina Genome Analyzer IIx (Illumina, San Diego, CA, USA).

### Transcriptome analysis

Reads that passed quality checks were aligned to the *F. vesca* genome using either a custom script or SeqMan NGen (DNASTAR version 2.3, Lasergene, Madison, WI, USA). *F. vesca* Genemark Hybrid version 1.1 was used for gene annotations. Gene candidates were identified in QSeq (DNASTAR) by making pairwise comparisons between high and low γ-D genotypes. Each pairwise comparison excluded genes less than 2 to 4-fold abundance when comparing high versus low γ-D genotypes. The genes remaining after these comparisons became gene candidates and were analyzed further by qRT-PCR.

A separate analysis was made using CLC Genomic Workbench software v6.5.0 (CLC Bio, Cambridge, MA, USA). The alignment parameters were adjusted to allow for expected variations in the octoploid genome relative to the diploid one (Minimum length fraction = 0.6; Minimum similarity fraction = 0.5; Maximum number of hits for a read = 5). RPKM value was used to normalize the expression data sets among individuals in the population. The data from Figure 3A overexpression data represent transcripts with a RPKM > 10 in at least one of the cultivars. A transcript was considered overexpressed when it was present at least 5 times more frequently in one genotype than the other. The functional classification of the differentially expressed genes was performed using MapMan ontology. The heatmap used for parents comparison was designed using Mapman software (Mapman version 3.6.0RC1,). A dot represents the log2 of the RPKM ratio of a transcript between the ‘Mara des Bois’ and ‘Elyana’ parents.

### qRT-PCR

cDNA templates for qRT-PCR were synthesized using the Impromtu II Reverse Transcriptase kit (Promega, Madison, WI, USA) according to the manufacturer’s protocol. The cDNA was diluted 1:10 prior to qRT-PCR analysis. All qRT-PCR reactions were run in 20 ul reactions using EvaGreen qPCR Mastermix-ROX (Applied Biological Materials Inc., Richmond, BC, Canada). Each reaction contained 10 ul 2x EvaGreen mastermix, 2 μl primer mix (2 uM each), 1 μl 1:10 diluted cDNA, and 7 μl DNase/RNase free water. All qRT PCR primers were designed using qRT primer design tools available online (idtdna.com), and designed to amplify fragments between 95 and 110 base pairs. Each primer-template combination was run with three technical replicates and three biological replicates. A conserved hypothetical protein (*FaCHP1*[42] was used as a housekeeping control (5′ TGCATATATCAAGCAACTTTACACTGA 3′ forward and 5′ ATAGCTGAGATGGATCTTCCTGTGA 3′ reverse). The qRT PCR was run on an Applied Biosystems StepOnePlus Real-Time PCR System using StepOne Software (v2.0) (Applied Biosystems, Foster City, CA, USA). The qRT-PCR data was analyzed using the comparative C_T_ method (ΔΔC_T_) following the manufacturer’s direction.

Candidate genes from the RNA-seq results were validated by qRT-PCR against templates from multiple genotypes, developmental stages, and/or environments. Candidates were initially validated using cDNA templates from all 16 genotypes included in the RNA-seq dataset. Further, candidates were tested for induction during ripening in ‘Elyana’ (only ripe fruits have detectable γ-D), and two environments with low or high γ-D production. The qRT-PCR sequences for *FaFAD1* (gene24414) were forward 5′ GTGCCCTTACTGATAACAAACG 3′ and reverse 5′ TCGCAACCAATCCCACTC 3′, for *CYTp450* (gene29958) forward 5′ ACCCAAAGGTCTATCACATGAC 3′ and reverse 5′ TGAGCTTCAGTTCCTAACCAC 3′, and for *FaFAD2* (gene22642) forward 5′ AACTGGTGTCTGGGTCATTG 3′ and reverse 5′ GAAAGGAGTGAAGGATCAGGC 3′.

### Cloning full length transcript for *FaFAD1*

The full length transcript for gene24414 was cloned using primer sequences guided by the transcriptome data. Primers were designed to include attb sites for Gateway (Invitrogen) cloning into pDONR222. Forward primer 5′ AAAAAGCAGGCTGCATGGGAGCCGATACCAAGTTCGAAGAG 3′ and a poly T reverse primer 5′ AGAAAGCTGGGTGTTTTTTTTTTTTTTTTTTTTTTTTTTTTTTTTTTTTT 3′ were initially used to obtain the full length transcript including 3′ UTR. A second reverse primer was designed from the cloned sequence up to and including the stop codon from the open reading frame (reverse 5′ AGAAAGCTGGGTGTTAGTTCCGGTACCAAAAAACACCTTTGGT 3′). A second round of PCR reconstructed full length attb sites using forward 5′ GGGGACAAGTTTGTACAAAAAAGCAGGCT 3′ and reverse 5′ GGGGACCACTTTGTACAAGAAAGCTGGGT 3′ primers. Standard PCR conditions were used with GoTaq polymerase (Promega) according to the manufacturer’s recommendations. The full length sequence, predicted protein translation, and physical map coordinates are provided in Additional file 2.

### Designing a functional molecular marker

A molecular marker for γ-D production was designed using the *F. vesca* genome sequence [26]. Primers were designed to amplify a 500 bp fragment from the 5′ end of gene24414 into the 5′ UTR: (forward) 5′ CGGGATTAATGGTTTTGTTGTTGACCGACC 3′ and (reverse) 5′ GTAGAAGAGAGAGACCAAGACGAG 3′. BFACT045 primers were previously shown to be linked to γ-D production and these primers were used as PCR controls forward 5′ CGACAAATGTAGTTGCTAGTCTTCTCA 3′ and reverse 5′ GAGGCAGAAGTGTTTTTCGTG 3′ [43]. The BFACT045 primers did not produce alleles that segregated with the γ-D phenotype in our populations as tested by capillary electrophoresis (data not shown). All PCR reactions (12.5 μl total volume) were run using GoTaq Hot Start polymerase (Promega) according to the manufacturer’s instructions. All thermocycler conditions were as follows: 94°C 4 min, followed by 25 cycles of 94°C 30 s, 56°C 30 s, 72°C 30 s, and with a final extension for 10 min at 72°.

The putative marker for gene24414 was tested against a subset of 17 progeny from the ‘Elyana’ x ‘Mara des Bois’ cross including parents, and two backcross populations to test segregation with the γ-D phenotype. Progeny were included in this analysis if they had three or more volatile samplings during the season to ensure accurate phenotyping of γ-D non-producers. Backcross population ‘Elyana’ x progeny 98 produced 26 progeny suitable for analysis, and backcross population ‘Mara des Bois’ x progeny 98 had 22 progeny suitable for analysis. Other octoploids suitable for consideration as breeding parents and/or for this study were also tested for marker cosegregation with the γ-D phenotype. These genotypes included ‘Radiance’, ‘Deutsch Evern’, ‘Festival’, ‘LF9’ (a self-pollination of ‘Festival’), ‘Albion’, ‘Winterstar’™ (‘FL 05-107’), ‘Mieze Schindler’, ‘Sweet Charlie’, and ‘Winter Dawn’.

A selection of wild, octoploid genotypes were also tested for the presence of the gene24414 marker, but were only included in this study if a minimum of three volatile samplings were performed. These genotypes are listed here by their Germplasm Resources Information Network Plant Inventory (PI) number and include accessions 236579, 612323, 612495, 612498, and 612499.

### Statistical marker analysis

Chi-square analysis was performed on marker and volatile data for all genotypes with at least three separate harvests during a season. Due to a strong environmental component, three harvests were necessary to provide the strongest evidence for accurately identifying a γ-D non-producer.

### SSR marker design

A 50 kb region of the genome surrounding gene24414 was downloaded using the Strawberry Genome Browser at the Genome Database for Rosaceae (rosaceae.org). The web version of BatchPrimer3 [44] was used to search for Simple Sequence Repeats (SSRs) near gene24414. Primers were designed to flank a dinucleotide repeat approximately 11 kb from gene24414. The primer pair (forward 5′ TGTAAAACGACGGCCAGTGAAGAAGATGACACTAGGGACGAGGAAG 3′ and reverse 5′ GTTCTATGTGAGAACATGGGAAGAAACATGAC 3′ with fluorescently labeled 5′ 6FAM-TGTAAAACGACGGCCAGT 3′) exhibited variation between ‘Elyana’ and ‘Mara des Bois’ and segregation in the fifteen progeny tested. These primers were used for allele detection during capillary electrophoresis as previously described [44].

### Availability of supporting data

The RNAseq reads have been deposited into the NCBI Short Read Archive and are accessible under project SRP039356 (http://www.ncbi.nlm.nih.gov/sra/?term=SRP039356). Data represent all reads from parental lines (‘Mara des Bois’ and ‘Elyana’) as well as reads from individual F1 plants.

## Competing interests

The authors declare that they have no competing interests.

## Authors’ contributions

AHC collected and prepared fruit samples for GCMS, isolate RNA for libraries, analyzed transcript reads, analyzed GCMS results, performed qPCR validations and structural genomic analyses. JP performed computational analyses on RNAseq data. AP performed GCMS analysis, provided interpretations and assisted in preparation of relevant areas of the manuscript. JB provided technical expertise with GCMS. VMW performed initial genetic crosses, raised progeny, collected fruit and assisted in manuscript preparation. KMF conceived the concept of the study, supervised and coordinated experiments, and prepared the manuscript. All authors have read and approved the manuscript.

## Supplementary Material

Additional file 1: Figure S1 An overview of the process used to identify candidate genes through analysis of bulk segregation of transcripts corresponding to a flavor volatile. Click here for file

Additional file 2 **The DNA and protein sequences of *FAD1.***
Click here for file
