# Supplementary material for: Identification of a strawberry flavor gene candidate using an integrated genetic-genomic-analytical chemistry approach
Source: BMC Genomics. 2014 Apr 17;15:217. doi: 10.1186/1471-2164-15-217 (PMC4023330; doi:10.1186/1471-2164-15-217)
Supplement: Additional file 1: Figure S1 — An overview of the process used to identify candidate genes through analysis of bulk segregation of transcripts corresponding to a flavor volatile. [file 1471-2164-15-217-S1.pdf]

Additional File 1.

# Flavor Candidates through Polyploid Transcript Homogenization

1. Use GC/MS to identify presence/absence of fruit volatiles in two cultivars

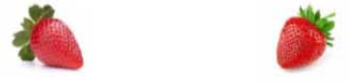

| | | |
|---|---|---|
| Volatile A | present (+) | not detected(--) |
| Volatile B | not detected | present |

2. Cross lines exhibiting different volatile profiles, then score progeny for the volatile.

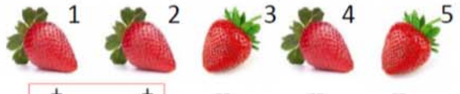

| Progeny | 1 | 2 | 3 | 4 | 5 |
|---|---|---|---|---|---|
| Volatile A | + | + | -- | -- | -- |
| Volatile B | -- | + | + | + | -- |

3. Sequence fruit RNA from individual parents and progeny

| | | | | | |
|---|---|---|---|---|---|
| Transcript 001 | + | + | -- | -- | -- |
| Transcript 002 | -- | + | -- | + | + |
| Transcript 003 | + | -- | -- | -- | -- |

4. Computationally merge transcriptome data into "producers" and "non-producers" to reveal candidates

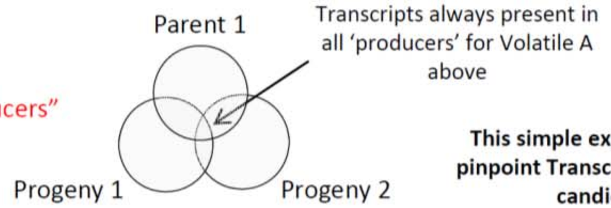


**This simple example would pinpoint Transcript 001 as the candidate**

**When many plant transcriptomes are merged based on presence / absence of a volatile, the candidate list decreases rapidly, as transcripts not relevant homogenize and expose those that are related to the process.**

5. Validation. The previous experiments test presence / absence in genotypes and their inheritance. Where else do we see presence / absence in volatile production? Do transcript candidates match those patterns?

**Accumulation with ripening**

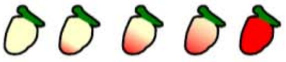

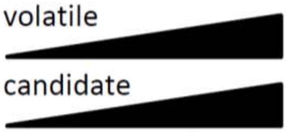


**PASS!**

**Presence/absence with environment**

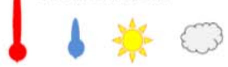

| | | | | |
|---|---|---|---|---|
| volatile | + | -- | + | -- |
| candidate | + | -- | + | -- |

**PASS!**

**Presence/absence in other cultivars**

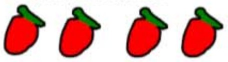

| | | | | |
|---|---|---|---|---|
| volatile | -- | -- | + | -- |
| candidate | -- | -- | + | -- |

**PASS!**

CONCLUSION: The use of merged data sets between producers, comparison against non-producers, and validation by correlation through multiple developmental, environmental and genetic tests indicates a high likelihood of relevance between the candidate and the volatile.
