# Supplementary material for: Identification of a strawberry flavor gene candidate using an integrated genetic-genomic-analytical chemistry approach
Source: BMC Genomics. 2014 Apr 17;15:217. doi: 10.1186/1471-2164-15-217 (PMC4023330; doi:10.1186/1471-2164-15-217)
Supplement: Additional file 2 — The DNA and protein sequences of FAD1. [file 1471-2164-15-217-S2.pdf]

Additional File 2.

>24414 consensus

ATGGGAGCCGATACCAAGTTCGAAGAGCAAATAAGGACACGTCAAAGGCTGCCGGTTTCGACTCCGCCATTCACTCT
AAGCCAACTCAAGAAAGCCATACCTCCACATTGCTTCAAGCGCTCTCTCCTCCGCTCCTTCTCCTATGTCATCTACG
ACCTCGTCTTGGTCTCTCTCTTCTACCACATCGCCACCTCTTACTTCCACCTCATCCCTCATCCCCTCTCCTACATC
GCATGGCCAGTCTATTGGATTCTCCAAGGCTGCACCCTCACCGGCGTGTGGGTCATCGCACACGAGTGTGGGCACCA
CGCCTTCAGTGACTACCAGCTCTTAGACGACGTCATTGGCCTTGTTCTTCACTCAGCTCTGTTGGTCCCTTACTTCT
CGTGGAAATACAGTCACAGACGCCACCATTCCAACATCGGATCGATGGAGAGAGATGAAGCCTTTGTTCCTAAACCC
AAATCTAAGATCTCATGGTACAACAAATACTTCAACAATCCACCAGGCAGGTTGATCACTATTATCTTCACCCTCAC
CCTCGGTTGGCCGTTGTACTTGGCATTTAACGTCTCCGGTCGACCCTACGACCGTTTCGCCTGCCACTATGACCCTT
ACAGTCCCATATTTTCCGACCGAGAAAGCCTTCAAATATATATCTCCGATCTCGGAATTTTAGTCACTGCTTCTGTA
CTCTACCGCCTTGCAATGGCTAAGGGATTGACTTGGCTCGTACGTGTTTATGGGGTGCCCTTACTGATAACAAACGG
GTTTCTTGTGTTGATCACATATTTGCAGCACACACACCCTTCACTGCCACACTACGACTCGTCGGAGTGGGATTGGT
TGCGAGGAGCTTTGTCGACCGTGGATAGAGACTGCGGGGTGCTCCATAAGGTTTTCCATAATATTACAGACACACAT
GTTGTTCATCATCTCTTCTCTACGATTCCACATTATAATGCAATGGAGGCCACAACAGCAGTGAAGCCTATATTGGG
AGATTACTATGGTTTTGATGGGACTCCGATTTACAAGGCTTTGTGGAGGGAGGCTAGGGAGTGCCTTTATGTTGAGC
CGGATGAAGATGCTCCTAATACCAAAGGTGTTTTTTGGTACCGGAACTAA

>24414 consensus translation

MGADTKFEEQIRTRQRLPVSTPPFTLSQLKKAIPPHCFKRSLLRSFSYVIYDLVLVSLFYHIATSYFHLIPHPLSYI
AWPVYWILQGCTLTGVWVIAHECGHHAFSDYQLLDDVIGLVLHSALLVPYFSWKYSHRRHHSNIGSMERDEAFVPKP
KSKISWYNKYFNNPPGRLITIIFTLTLGWPLYLAFNVSGRPYDRFACHYDPYSPIFSDRESLQIYISDLGILVTASV
LYRLAMAKGLTWLVRVYGVPLLITNGFLVLITYLQHTHPSLPHYDSSEWDWLRGALSTVDRDCGVLHKVFHNITDTH
VVHHLFSTIPHYNAMEATTAVKPILGDYYGFDGTPIYKALWREARECLYVEPDEDAPNTKGVFWYRN-

LG3:31,112,418..31,114,643
scf0513029:129621..131846
